# Disparities in COVID-19 Disease Incidence by Income and Vaccination Coverage — 81 Communities, Los Angeles, California, July 2020–September 2021

**DOI:** 10.15585/mmwr.mm7226a5

**Published:** 2023-06-30

**Authors:** John M. Masterson, Michael Luu, Kai B. Dallas, Lauren P. Daskivich, Brennan Spiegel, Timothy J. Daskivich

**Affiliations:** ^1^Department of Urology, Cedars-Sinai Medical Center, Los Angeles, California; ^2^Department of Biostatistics, Cedars-Sinai Medical Center, Los Angeles, California; ^3^Division of Urology, City of Hope, Duarte, California; ^4^Los Angeles County Department of Health Services, Los Angeles, California; ^5^Department of Medicine, Cedars-Sinai Medical Center, Los Angeles, California; ^6^Cedars-Sinai Center for Outcomes Research and Education, Cedars-Sinai Medical Center, Los Angeles, California.

COVID-19 has disproportionately affected socially vulnerable communities characterized by lower income, lower education attainment, and higher proportions of minority populations, among other factors (*1*–*4*). Disparities in COVID-19 incidence and the impact of vaccination on incidence disparities by community income were assessed among 81 communities in Los Angeles, California. Median community vaccination coverage and COVID-19 incidence were calculated across household income strata using a generalized linear mixed effects model with Poisson distribution during three COVID-19 surge periods: two before vaccine availability (July 2020 and January 2021) and the third after vaccines became widely available in April 2021 (September 2021). Adjusted incidence rate ratios (aIRRs) during the peak month of each surge were compared across communities grouped by median household income percentile. The aIRR between communities in the lowest and highest median income deciles was 6.6 (95% CI = 2.8–15.3) in July 2020 and 4.3 (95% CI = 1.8–9.9) in January 2021. However, during the September 2021 surge that occurred after vaccines became widely availabile, model estimates did not identify an incidence disparity between the highest- and lowest-income communities (aIRR = 0.80; 95% CI = 0.35–1.86). During this surge, vaccination coverage was lowest (59.4%) in lowest-income communities and highest (71.5%) in highest-income communities (p<0.001). However, a significant interaction between income and vaccination on COVID-19 incidence (p<0.001) indicated that the largest effect of vaccination on disease incidence occured in the lowest-income communities. A 20% increase in community vaccination was estimated to have resulted in an additional 8.1% reduction in COVID-19 incidence in the lowest-income communities compared with that in the highest-income communities. These findings highlight the importance of improving access to vaccination and reducing vaccine hesitancy in underserved communities in reducing disparities in COVID-19 incidence.

Eighty-one communities in Los Angeles with available vaccination and incidence data (total population = 5,083,093; median = 47,450) were included in the analysis (Supplementary Table 1, https://stacks.cdc.gov/view/cdc/129934). Community-level COVID-19 vaccination coverage and incidence data from March 2020 through September 2021 were obtained from the Los Angeles Times COVID-19 data repository, which is populated with California Department of Public Health data.* COVID-19 incidence data from this repository and sociodemographic data from the U.S. Census Bureau^†^ were available by community name within Los Angeles County. Vaccination coverage data were available by zip code. Los Angeles Times COVID-19 incidence and vaccination data were linked to census data using zip codes as a common identifier.

A generalized linear mixed effects model with a Poisson distribution was used to estimate COVID-19 incidence and, separately, vaccination coverage across strata of median community household income. Covariates in the model included percentage of persons in each community who had completed the primary COVID-19 vaccination series^§^ and the following community characteristics: percentage of persons who were 1) aged ≥65 years; 2) male or female; 3) non–U.S.-born; 4) non-Hispanic White, non-Hispanic Black or African American, or Hispanic or Latino; 5) who had completed at least high school; and 6) who had no health insurance; and the average number of persons residing in each household. Time (number of months since data collection began in March 2020) and vaccination coverage were included in the model as polynomial splines to allow flexibility in estimating the nonlinear effects of time and vaccination coverage on COVID-19 incidence. Interaction terms along with main effects for median income and vaccination coverage, and median income and time in months were included to adjust for differential effects of median income on COVID-19 incidence across levels of vaccination coverage and time.^¶^ Unadjusted** and adjusted IRRs with predicted marginal effects for COVID-19 incidence were calculated across percentiles of median community household income during July 2020, January 2021, and September 2021. The aIRRs during the peak month of each surge were compared across income strata using the Wald’s test with p-values adjusted using Tukey’s method for multiple comparisons. Median community vaccination coverage and reduction in COVID-19 incidence associated with a 20% increase in community vaccination coverage in September 2021 were estimated independently with the multivariable mixed effects Poisson models and were compared across income strata using pairwise contrasts of Wald’s test, with p-values adjusted using Tukey’s method for multiple comparisons. Statistical tests were performed using R software (version 4.1.1; R Foundation). All tests were two‐sided with a significance threshold of p<0.05. This study was deemed exempt from IRB review by the Cedars-Sinai Institutional Review Board.

COVID-19 incidence was significantly higher in communities with lower median household income than in those with higher median household income during both July 2020 (aIRR = 6.6, 95% CI = 2.8–15.3) and January 2021 (aIRR = 4.3; 95% CI = 1.8–9.9) (Figure) (Table 1).^††^ In September 2021, however, incidence was not significantly different across communities irrespective of median household income (aIRR = 0.8; 95% CI = 0.35–1.86). By September 2021, higher median household income was associated with higher community vaccination coverage across all percentiles of income, with median vaccination coverage of 59.4% (95% CI = 57.6%–61.2%) for the lowest- and 71.5% (95% CI = 68.5%–74.4%) for the highest-income communities (p<0.001) (Supplementary Figure, https://stacks.cdc.gov/view/cdc/129936) (Supplementary Table 2, https://stacks.cdc.gov/view/cdc/129935). A significant interaction was observed between median household income and vaccination coverage on COVID-19 incidence (p<0.001). Within each income stratum, vaccination coverage was inversely associated with COVID-19 incidence; however, the effect was largest in the lowest-income communities. A 20% increase in community vaccination coverage was predicted to result in an additional 8.1% (95% CI = 7.7%–8.4%) reduction in COVID-19 incidence in the lowest-income communities compared with the highest-income communities (p<0.001) (Table 2).

**FIGURE:**
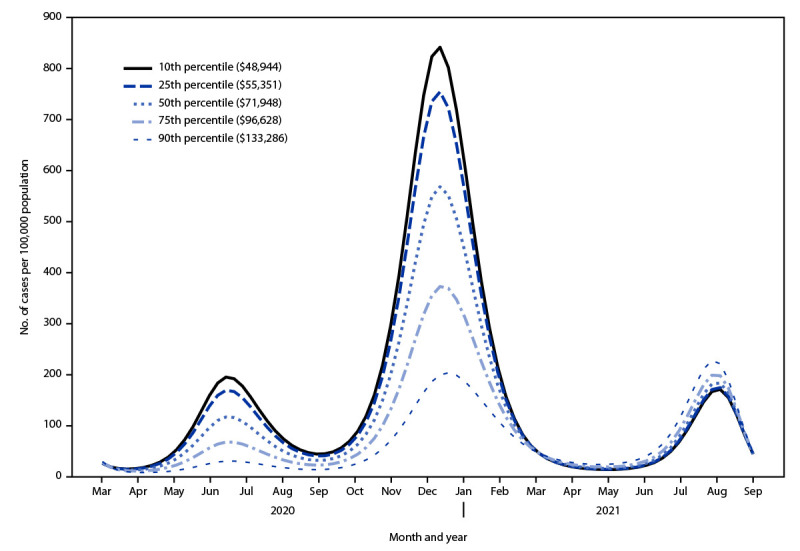
Estimated COVID−19 incidence,* by median community income percentile^†^^,^^§^ — 81 communities, Los Angeles, California, March 2020–September 2021 * Cases per 100,000 population. Incidence was estimated across median community income strata using the multivariable Poisson mixed effects model corrected for community-level median vaccination coverage, age, sex, non–U.S.-born status, race, ethnicity, education level, number of persons per household, insurance status, and interaction terms for income by vaccination and income by time in months since data collection began in March 2020. ^†^ In U.S. dollars. p<0.001 for all comparisons of COVID-19 incidence between median community income strata in July 2020 and January 2021 (independently) using Wald’s test adjusted for multiple comparisons. ^§^ In U.S. dollars. p<0.05 for all comparisons of COVID-19 incidence between median community income strata in September 2021 using Wald’s test adjusted for multiple comparisons.

**TABLE 1:** Comparison of COVID-19 unadjusted and adjusted incidence rate ratios,* by median community income percentile^†^ — 81 communities, Los Angeles, California, July 2020, January 2021, and September 2021

Median community income percentile^§^ comparison	IRR (95% CI), by month and year					
	Jul 2020^§^		Jan 2021^§^		Sep 2021	
	Unadjusted	Adjusted	Unadjusted	Adjusted	Unadjusted	Adjusted
10th vs. 25th	1.14 (1.11–1.17)	1.15 (1.08–1.23)	1.09 (1.06–1.12)	1.12 (1.05–1.19)	1.03 (0.99–1.06)	0.98 (0.92–1.05)
10th vs. 50th	1.59 (1.43–1.77)	1.67 (1.33–2.11)	1.37 (1.23–1.52)	1.49 (1.18–1.87)	1.10 (0.98–1.22)	0.94 (0.75–1.18)
10th vs. 75th	2.63 (2.11–3.28)	2.91 (1.80–4.69)	1.92 (1.54–2.40)	2.28 (1.42–3.68)	1.22 (0.98–1.52)	0.88 (0.54–1.41)
10th vs. 90th	5.52 (3.74–8.16)	6.61 (2.84–15.38)	3.18 (2.16–4.69)	4.30 (1.85–9.99)	1.41 (0.96–2.09)	0.79 (0.34–1.84)

**Abbreviation:** IRR = incidence rate ratio.

* IRRs were estimated from multivariable Poisson mixed effects model for COVID-19 incidence. Univariable (unadjusted) models included time in months since data collection began in March 2020, income, and the interaction of income by time. Multivariable (adjusted) models included community-level median vaccination coverage, age, sex, non–U.S.-born status, race, ethnicity, education level, number of persons per household, insurance status, and interaction terms for income by vaccination and income by time.

**^†^** Median community income percentiles (in U.S. dollars): 10th = $48,944; 25th = $55,351; 50th = $71,948; 75th = $96,628; and 90th = $133,286.

^§^ p<0.001 for IRR comparisons between median community income strata at the specified time point using the Wald’s test adjusted for multiple comparisons.

**TABLE 2:** Predicted absolute reduction* in COVID-19 incidence rate ratios associated with a 20% increase in community primary series coverage^†^ — 81 communities, Los Angeles, California, September 2021

Median community income percentile, (USD)	Absolute reduction in COVID-19 incidence,^§,¶^ % (95% CI)
90th ($133,286)	Ref
75th ($96,628)	−3.6 (−3.2 to −4.0)
50th ($71,948)	–5.9 (−5.7 to −6.2)
25th ($55,351)	−7.5 (−7.2 to −7.8)
10th ($48,944)	−8.1 (−7.7 to −8.4)

**Abbreviations:** Ref = referent group; USD = U.S. dollars.

* Reductions in incidence rate ratios estimated from the multivariable Poisson mixed effects model corrected for community-level median vaccination coverage, age, sex, non–U.S.-born status, race, ethnicity, education level, number of persons per household, insurance status, and interaction terms for income by vaccination and income by time in months since data collection began in March 2020.

^†^ Receipt of 1 dose of Janssen (Johnson & Johnson) vaccine or 2 doses of Pfizer-BioNTech or Moderna vaccines.

^§^ COVID-19 vaccination and incidence data were obtained from the Los Angeles Times COVID-19 data repository (https://www.latimes.com/projects/california-coronavirus-cases-tracking-outbreak/). Sociodemographic data was obtained from 2019 U.S. Census Bureau data.

^¶^ p<0.001 for pairwise comparisons versus the 90th percentile median community income using the Wald’s test adjusted for multiple comparisons.

## Discussion

This study adds to the body of evidence showing the disproportionate impact of COVID-19 on the lowest-income communities early in the pandemic and the impact of vaccination in reducing these disparities. These disparities were mitigated during the third pandemic surge, after COVID-19 vaccines became widely available. Although vaccination coverage was inversely associated with disease incidence during the third surge in all income groups, the estimated impact of vaccination on COVID-19 incidence was largest in the lowest-income communities, despite lower overall vaccination coverage in these communities. The higher impact of vaccination in those communities might be due to the higher risk for SARS-CoV-2 exposure in lower-income communities, potentially related to higher population density, more use of public transportation, and increased likelihood of working in service industries in which remote work might not be feasible (*5*). Higher COVID-19 incidence in lower-income communities might also have contributed to higher levels of postinfection immunity before the third surge, with 17% of the population in the lowest-income communities having received a positive COVID-19 test result before the third surge compared with only 4% in the highest-income communities.

Vaccination coverage differed by income despite public health programs to enhance access to vaccination in lower-income communities. Efforts in California included allocating 40% of vaccination appointments to communities in the lowest quartile of the California Healthy Places Index (HPI) (https://www.healthyplacesindex.org/) early in the vaccine rollout (i.e., March 2021) (*6*). HPI reflects 25 community characteristics using data related to household income, education level, health care access, housing, neighborhoods, clean environment, transportation, and social environment. California’s zip codes (≥1,650) were stratified by the HPI Index (*7*). All communities in the lowest decile of median income in this study were also in the lowest quartile of HPI. This vaccine allocation effort and other factors likely contributed to the relatively narrow range of estimated adult primary series vaccination coverage rates (59.4%–71.5%) observed across income strata in Los Angeles County communities at the time of the September 2021 surge.

Efforts to improve vaccination access and vaccine confidence are needed to mitigate income-related vaccination disparities (*8*). Racial, ethnic, income, birth origin, and education inequalities in adult routine vaccination are longstanding, highlighting the continued need to build vaccine confidence for COVID-19 and routine immunization (*9*).

The findings in this report are subject to at least three limitations. First, these data sets do not include person-level data to enable direct estimation of the impact of individual vaccination on COVID-19 incidence by income, which precluded accounting for individual immunity acquired from previous infection. Second, it was not possible to adjust for differential access to or use of testing between communities over time, which has potential to inflate or dampen observed disaparities. Finally, these results might not be generalizable outside Los Angeles or to other pandemic waves, the latter due to differences in vaccine effectiveness among different COVID-19 variants.

The COVID-19 pandemic has highlighted the impact of social determinants of health on health disparities (*10*). Future planning is needed to ensure readiness to quickly implement strategies to mitigate disparities during pandemics affecting lower-income communities while vaccines are being developed, including efforts to improve access to vaccination and vaccine confidence in disproportionately affected communities. Reducing barriers to vaccination in lower-income communities, including providing updated (bivalent) COVID-19 vaccine boosters, is critical to reducing disparities in disease impact, and decreasing COVID-19–related illness in the United States.

**Summary**

**What is already known about this topic?**

The COVID-19 pandemic disproportionately affected lower-income communities.

**What is added by this report?**

In 81 communities in Los Angeles, California, COVID-19 incidence during two surges before vaccine availability (July 2020 and January 2021) was higher in lower-income communities compared with higher-income communities. During the first surge after vaccines became available (September 2021), a disparity in COVID-19 incidence between the highest- and lowest-income communities was not observed. The impact of vaccination on COVID-19 incidence was highest in the lowest-income communities despite their lower vaccination coverage.

**What are the implications for public health practice?**

Addressing barriers to vaccination within lower-income communities is critical to reducing disparities in disease incidence and COVID-19–related illness in the United States.

## Footnotes

* Summary: https://www.latimes.com/projects/california-coronavirus-cases-tracking-outbreak/; primary data: https://github.com/datadesk/california-coronavirus-data (Accessed October 1, 2022).

† https://www.census.gov/quickfacts/fact/table/US/PST045221 (Accessed October 1, 2022).

§ Receipt of 1 dose of Janssen (Johnson & Johnson) vaccine or 2 doses of Pfizer-BioNTech or Moderna vaccines.

¶ The interaction between income and time was included to account for differential effects of median income on COVID-19 incidence at different time points. Without this interaction term, it would be assumed that the effect of median income on COVID-19 incidence is independent of time and hence fixed across the analyzed time points. The interaction between vaccination and time was not included in the model because widespread vaccination did not occur until the end of the time horizon of the study and was therefore relevant only to the final surge in September 2021.

** Univariable (unadjusted) models included time in months relative to the start of data collection (March 2020), income, and the interaction of income by time.

†† A significant interaction was identified between income and time (p<0.001) in the mixed effects model predicting COVID-19 incidence, in which the effects of income on COVID-19 incidence was dependent on time.
